# 盐酸埃克替尼一线治疗晚期肺腺癌的临床疗效观察

**DOI:** 10.3779/j.issn.1009-3419.2013.07.06

**Published:** 2013-07-20

**Authors:** 新杰 杨, 卉 张, 娜 秦, 曦 李, 靖颖 农, 嘉林 吕, 羽华 吴, 权 张, 树才 张

**Affiliations:** 101149 北京，首都医科大学附属北京胸科医院/北京市结核病胸部肿瘤研究所肿瘤内科 Department of Medical Oncology, Beijing Chest Hospital, Capital Medical University/Beijing Tuberculosis and Thoracic Tumor Research Institute, Beijing 101149, China

**Keywords:** 肺肿瘤, 盐酸埃克替尼, 一线治疗, 腺癌, Lung neoplasms, Icotinib hydrochloride, First-line therapy, Adenocarcinoma

## Abstract

**背景与目的:**

分子靶向治疗药物盐酸埃克替尼治疗复治晚期非小细胞肺癌具有较好的疗效和安全性。本文观察盐酸埃克替尼一线治疗晚期肺腺癌的近期疗效和毒副反应。

**方法:**

对2011年8月-2012年11月间收治的56例初治晚期肺腺癌患者，口服盐酸埃克替尼125 mg，每日3次，评价近期疗效和不良反应。

**结果:**

56例肺腺癌患者的客观有效率为46.4%（26/56），疾病控制率为78.6%（44/56）。20例患者进行了*EGFR*基因突变检测，18例患者*EGFR*基因突变阳性，*EGFR*突变阳性患者的客观有效率为66.7%（12/18），疾病控制率为94.4%（17/18）。不吸烟、*EGFR*基因突变阳性和出现皮疹的患者客观有效率高于吸烟、*EGFR*基因野生型和突变情况未知、未出现皮疹的患者（*P* < 0.05）。治疗相关毒副反应主要为轻度皮疹（28.5%）和腹泻（12%）。

**结论:**

盐酸埃克替尼一线治疗晚期肺腺癌疗效肯定，不良反应轻微，对于*EGFR*突变阳性患者有效率更高。

## Abstract (en)

**Background and objective:**

It has been proven that icotinib hydrochloride, as a molecule targeted drug, can be safely and efficiently used to treat advanced non-small cell lung cancer (NSCLC) for second-line or third-line. This research was aimed to investigate the efficacy and toxicity of icotinib hydrochloride as the first-line therapy for pulmonary adenocarcinoma.

**Results:**

Among the 56 patients, the tumor objective response rate (ORR) and disease control rate (DCR) was 46.4% (26/56) and 78.6% (46/56), respectively. Among the 20 patients with EGFR analyses, 18 patients were positive for a mutation, ORR was 66.7% (12/18), DCR was 94.4% (17/18) respectively. The ORR with no history of smoke. *EGFR* positive mutation and appearance of rash were significantly higher than those with smoker, wild type *EGFR*, no information about EGFR and no appearance of rash (*P* < 0.05). The most common drug-related adverse events were mild skin rash (28.5%) and diarrhea (12%).

**Conclusion:**

Single agent treatment with icotinib hydrochloride is effective and tolerable in first-line therapy for pulmonary adenocarcinoma, especially with *EGFR* mutation.

近年来，肺癌发病率逐年上升，是导致癌症死亡的主要原因^[1]^。非小细胞肺癌（non-small cell lung cancer, NSCLC）中仅不到1/3的患者为早期发现，可手术切除^[2]^。较之于最佳支持治疗，传统的化疗仅将晚期NSCLC患者的1年生存率从20%提高至29%^[3]^，体力状态差的患者更是无法从传统化疗中获益。近年来几项大型Ⅲ期临床研究^[4-8]^报道，人表皮生长因子酪氨酸激酶抑制剂（epidermal growth factor receptor tyrosine kinase inhibitors, EGFR-TKIs）吉非替尼和厄洛替尼在晚期肺癌（尤其是亚裔、女性、腺癌、不吸烟）患者中具有较好的临床疗效和安全性。盐酸埃克替尼（icotinib hydrochloride）是一种高效特异性的EGFR-TKI，是我国第一个具有自主知识产权的小分子靶向抗癌新药，与吉非替尼和厄洛替尼相比，在化学结构、分子作用机理、疗效等方面类似，但具有更好的安全性，适用于晚期NSCLC患者的治疗^[9-12]^。但目前国内尚无关于盐酸埃克替尼一线治疗晚期肺腺癌的相关报道。我科室对一线应用盐酸埃克替尼治疗的56例晚期肺腺癌患者进行了近期疗效及相关药物毒副反应的总结分析。

## 1 资料与方法

### 1.1 临床资料

选择2011年8月-2012年11月我科室收治的盐酸埃克替尼一线治疗的晚期肺腺癌患者56例，所有患者均经细胞学或组织学证实并有完整的临床资料，至少具有1个可测量病灶，治疗前患者的血常规及肝肾功能均正常。其中男性21例，女性35例; 年龄39岁-87岁，中位年龄67.88岁; 根据美国东部肿瘤协作组（ECOG）体能状态评分，0分-2分46例，3分-4分10例; 吸烟13例，轻度吸烟7例，不吸烟36例; 按2002年美国癌症研究联合会癌症分期手册（第六版）NSCLC分期标准，Ⅲb期3例，Ⅳ期53例; 共有20例患者应用突变富集液相芯片法进行了*EGFR*基因突变检测，其中外显子19缺失突变10例，外显子21错义突变8例，野生型2例（表 1）。

**1:** 56例肺腺癌患者临床特征与疗效分析 Analysis of the characteristics and response of 56 patients

Characteristics	*n* (%)	CR	PR	SD	PD	ORR (%)	*P*	DCR (%)	*P*
Gender							0.785		0.108
Male	21 (37.5)	0	9	5	7	42.9		66.7	
Female	35 (62.5)	0	17	13	5	48.6		85.7	
Age (yr)							0.999		0.999
< 70	26 (46.4)	0	12	8	6	46.2		76.9	
≥70	30 (53.6)	0	14	10	6	46.7		80.0	
PS (ECOG)							0.087		< 0.001
0-2	46 (82.1)	0	24	18	4	52.2		91.3	
3-4	10 (17.9)	0	2	0	8	20.0		20.0	
Smoker							0.038		0.017
Never	36 (64.3)	0	20	13	3	55.6		86.1	
Light	7 (12.5)	0	4	1	2	57.1		71.4	
Non-light	13 (23.2)	0	2	4	7	15.4		46.2	
Stage							0.592		0.522
Ⅲb	3 (5.4)	0	2	0	1	66.7		66.7	
Ⅳ	53 (94.6)	0	24	18	11	45.3		79.2	
*EGFR* mutation							0.010		0.129
19 deletion	10 (17.9)	0	9	1	0	90.0		100.00	
L858R	8 (14.3)	0	3	4	1	37.5		87.5	
Wild type	2 (3.6)	0	0	1	1	0.00		50.0	
Unknown	36 (64.3)	0	14	12	10	38.9		72.2	
Rash							0.043		0.012
No	40 (71.4)	0	15	13	12	37.5		70.0	
Yes	16 (28.6)	0	11	5	0	68.8		100.00	
Diarrhea							0.358		0.666
No	50 (89.3)	0	25	13	12	50.0		76.0	
Yes	6 (10.7)	0	1	5	0	16.7		100.00	
PS: performance status; EGFR: epidermal growth factor receptor; CR: complete response; PR: partial response; SD: stable response; PD: progressive disease; ORR: objective response rate; DCR: disease control response.									

### 1.2 治疗方法

口服盐酸埃克替尼125 mg，每日3次，持续用药直至肿瘤进展或出现不可耐受的不良反应。治疗4周后进行近期疗效及不良反应评价。停用盐酸埃克替尼后，根据患者的体力状态评分（performance status, PS）和意愿给予化疗、放疗或最佳支持治疗。

### 1.3 评价标准

吸烟指数为0定义为不吸烟，吸烟指数大于0小于等于20包/年定义为轻度吸烟，吸烟指数大于20包/年定义为吸烟。根据实体瘤疗效评价标准（RECIST）1.1对肿瘤客观缓解率进行评价，分为完全缓解（complete response, CR）、部分缓解（partial response, PR）、疾病稳定（stable disease, SD）和疾病进展（progressive disease, PD）。客观缓解率（objective response rate, ORR）是指CR+PR患者占全组患者的百分率，疾病控制率（disease control rate, DCR）是指CR+PR+SD患者占全组患者的百分率。按照美国国立癌症研究所制订的不良反应评价标准（NCI-CTC 3.0版）不良反应分为0级-V级。

### 1.4 统计学方法

采用SPSS 21.0统计软件进行统计分析，疗效和临床特征分析采用*Fisher’*精确检验和*Logistic*回归分析。*P* < 0.05为差异有统计学意义。

## 2 结果

### 2.1 近期疗效

所有患者均可评价疗效。56例患者中，无CR病例，PR 26例（46.4%），SD 18例（32.2%），PD 12例（21.4%），ORR为46.4 %，DCR为78.6%（表 1）。20例*EGFR*突变检测患者中，18例患者*EGFR*基因突变阳性，其中无CR病例，PR 12例（66.7%），SD 5例（27.8%），PD 1例（5.6%），ORR为66.7%，DCR为94.4%。

### 2.2 症状缓解情况

全组有31例（55.4%）患者在治疗后有不同程度的症状缓解，主要缓解的症状为咳嗽、喘憋、疼痛、声嘶等; 23例（41.1%）患者PS评分得到改善。服药2天至14天症状改善，中位起效时间为10天。

### 2.3 临床特征与盐酸埃克替尼治疗疗效的相关性

56例患者中，是否吸烟、*EGFR*基因突变状态及有无皮疹与盐酸埃克替尼治疗的ORR相关，差异有统计学意义（*P*=0.038, *P*=0.010, *P*=0.043），PS评分、是否吸烟及有无皮疹与盐酸埃克替尼治疗的DCR相关，差异有统计学意义（*P* < 0.001, *P*=0.017, *P*=0.012）（表 1）; 18例*EGFR*基因突变阳性患者中，除外显子19缺失突变患者ORR高于外显子21错义突变患者，差异有统计学意义外（*P*=0.043），其余包括性别、年龄、PS评分、是否吸烟、有无皮疹及腹泻单因素在内的亚组分析中均未发现疗效具有差异。多因素*Logistic*回归分析56例患者，只有吸烟与肿瘤客观缓解率相关（*P*=0.020, HR 17.772, 95%CI: 1.559-202.539）; 而在18例*EGFR*基因突变阳性的患者中，未发现疗效相关因素（*P* > 0.05）。

### 2.4 不良反应

56例患者中，治疗相关不良反应发生率为66.1%，其中多为轻度，仅有2例患者出现Ⅲ度以上不良反应，未因不可耐受毒副反应而退出治疗。无1例发生间质性肺炎，未发现药物相关性死亡。最常见的不良反应主要为皮疹28.6%（16/56）、腹泻10.7%（6/56）、指甲改变3.6%（2/56）、皮肤瘙痒8.9%（5/56）、胃部不适3.6%（2/56）、肝功能异常10.7%（6/56）（表 2）。

**2:** 不良反应 Adverse events

Adverse events	Grade (*n*=56)		rate (%)
	Ⅰ-Ⅱ	Ⅲ-Ⅳ	
Rash	15	1	28.6
Diarrhea	5	1	10.7
Nail changes	2	0	3.6
Pruritus	5	0	8.9
Stomatitis	2	0	3.6
ALT	4	0	7.2
AST	2	0	3.6

## 3 讨论

目前已有多项大型Ⅲ期临床试验结果报道^[4-8]^人EGFR-TKIs吉非替尼和厄洛替尼在晚期NSCLC患者的各线治疗中都显示出良好的临床疗效和安全性。盐酸埃克替尼作为我国自主研发的Ⅰ类新药，在上市前的Ⅲ期临床试验中（ICOGEN）显示出埃克替尼和吉非替尼均可明显改善NSCLC患者的生活质量，且埃克替尼总不良反应发生率低于吉非替尼^[13]^。李曦等^[14]^对埃克替尼治疗晚期NSCLC的疗效观察也证实了埃克替尼良好的临床疗效与安全性。

2009年，Mok等^[4]^在IPASS临床研究中，共有609例患者接受吉非替尼靶向治疗，ORR达43.0%，明显高于同期化疗组（32.2%），研究结果提示亚裔、腺癌、不吸烟及女性患者是靶向治疗的优势人群。基于IPASS研究结果，我科室回顾性分析了盐酸埃克替尼一线治疗晚期肺腺癌患者的临床疗效。在本研究中，56例肺腺癌患者接受盐酸埃克替尼靶向治疗，ORR为46.3%，DCR为78.6%，这一结果与IPASS研究相似，其原因可能与本研究人群均为腺癌，与IPASS研究人群相类似，都属靶向治疗的优势人群。在进一步的亚组分析中显示，盐酸埃克替尼的疗效与有无吸烟史、*EGFR*基因是否存在敏感突变和是否出现皮疹相关（*P*=0.038, *P*=0.010, *P*=0.043），这也与IPASS研究结果基本一致，但其中皮疹与客观疗效相关的结果却在同类临床研究中少有报道。但与本研究结果一致的是，盐酸埃克替尼在晚期NSCLC的二线及三线治疗中^[13]^，疗效与临床分期、性别、病理类型、吸烟史及ECOG评分相关，差异有统计学意义（*P* < 0.05），而其中出现皮疹的患者也显示出较好的临床疗效。

IPASS研究^[4]^中，存在*EGFR*基因敏感突变的患者一线接受吉非替尼治疗客观缓解率达71.2%，而无*EGFR*基因突变者仅为1.1%。后续报道的WJTOG3405^[5]^、NEJ002试验^[6]^及中国的OPTIMAL研究^[8]^等均显示出与IPASS研究相近的结果。基于这些临床研究结果，2010年美国和中国的肿瘤临床指南均明确指出，对于*EGFR*基因突变的晚期NSCLC患者，推荐一线可使用EGFR-TKIs治疗。本组56例患者中，有20例利用突变富集液相芯片法进行了*EGFR*基因突变检测，突变阳性率90%（18/20），明显高于IPASS研究中59.7%的突变率（261/437）及2012年ASCO报道的PIONEER研究^[15]^中51.4%（746/1, 450）的结果，其主要原因是由于本研究纳入的治疗病例均为一线接受靶向治疗的患者，按照治疗指南要求经检测*EGFR*基因突变阴性的患者大部已被排除在外，仅有2例*EGFR*野生型患者因为PS评分为3分-4分，无法耐受化疗而一线接受盐酸埃克替尼靶向治疗。本研究另有36例患者因肿瘤组织标本缺乏或过少无法满足检测要求而未能进行*EGFR*基因突变状态的检测，但因患者拒绝化疗或因PS评分3分-4分无法耐受化疗而一线接受盐酸埃克替尼靶向治疗。18例突变阳性的患者中有10例（55.6%）为外显子19缺失突变，8例（44.4%）为外显子21错义突变，这一比例也与IPASS研究相似（53.6%和42.5%）。本研究中存在*EGFR*基因突变的患者ORR为66.7%，DCR达94.4%，与之前报道的OPTIMAL研究^[8]^（ORR 83%，DCR 96%）、WJTOG3405研究^[5]^（ORR 62.1%，DCR 93.1%）及IPASS研究^[4]^（ORR 71.2%）非常接近，反映出盐酸埃克替尼一线治疗存在*EGFR*基因突变的患者，疗效与吉非替尼和厄洛替尼接近。进一步利用多因素*Logistic*回归分析发现所有56例肺腺癌患者仅有是否吸烟成为影响客观有效率的独立因素（*P*=0.020）。在18例*EGFR*基因突变阳性的患者中，却没有发现影响疗效的独立因素（*P* > 0.05），究其原因可能由于研究例数较少，而说服力不足，但也可能与本研究56例患者均为腺癌，具有较高*EGFR*基因突变率有关，根据PIONEER研究结果^[15]^推测，36例未行*EGFR*基因突变检测的患者至少50%可能为*EGFR*基因突变阳性，在突变患者占多数的条件下，未发现治疗疗效相关因素，这也间接反映出*EGFR*基因突变可能是决定盐酸埃克替尼治疗疗效的主要影响因素，但这一结果还需更大样本量的临床研究证实。

本研究中，存在外显子19缺失突变患者的ORR和DCR分别为90.0%和100%，均高于外显子21错义突变患者（37.5%和87.5%），两者在ORR上的差异具有统计学意义（*P*=0.043）。在盐酸埃克替尼的Ⅲ期临床试验中^[13]^，外显子19缺失突变与外显子21错义突变患者的ORR分别为88%比22%。另外一项在美国和欧洲进行的临床研究结果报道^[16]^，具有外显子19突变的患者比具有外显子21突变的患者无疾病进展时间和总生存时间更长（*P* < 0.000, 1）。这些似乎都显示出外显子19突变的人群更能从EGFR-TKI治疗中获益。但在I-CAMP^[17]^和WJTOG3405^[5]^临床研究结果中并未看到两者之间的疗效和生存差异。虽然本研究的远期生存数据尚不成熟，但近期疗效观察已显示出盐酸埃克替尼在外显子19缺失突变患者中的治疗优势，其中具体机制和原因尚不明确，还需更深入的基础研究和更大样本量的临床观察。

本研究中盐酸埃克替尼治疗相关毒副反应主要为皮疹、腹泻及转氨酶升高，其它少见的不良反应为轻度胃部不适、皮肤瘙痒、指甲改变等，以上不良反应的发生率及严重程度与ICOGEN的研究数据相似^[13]^。其发生率及皮疹的严重程度均低于吉非替尼^[4]^，更低于厄洛替尼^[8]^。未发生不可耐受的不良反应而退出治疗，无间质性肺炎发生，未发生药物相关性死亡，临床耐受性良好。

综上所述，盐酸埃克替尼一线治疗晚期肺腺癌近期疗效肯定，对于*EGFR*突变患者有效率更高，治疗相关的不良反应轻，临床治疗耐受性好，但因上市时间较短，远期生存资料尚未获得，还需要更多的大样本、前瞻性、多中心的临床研究验证其临床疗效。
